# Supplementary material for: High Glycolytic Activity Enhances Stem Cell Reprogramming of Fahd1-KO Mouse Embryonic Fibroblasts
Source: Cells. 2021 Aug 10;10(8):2040. doi: 10.3390/cells10082040 (PMC8392800; doi:10.3390/cells10082040)
Supplement: Supplementary file 1 [file cells-10-02040-s001.zip › cells-1307942-supplementary.pdf]

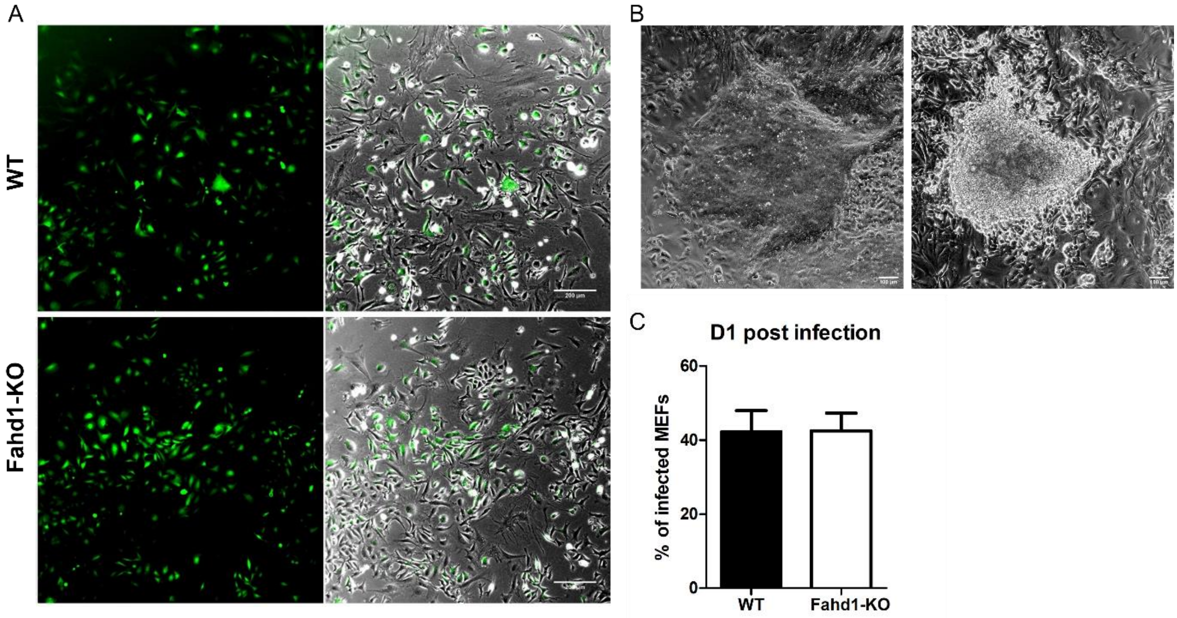

Figure S1: Similar transfection efficiency between WT and Fahd1-KO MEFs at day 1 post infection. A, Fluorescent and merged with phase contrast images of WT and Fahd1-KO MEFs showing fluorescent cells D1 after infection with the reprogramming factors; scale bar, 200 µm. B, Phase contrast images of the iPSC like colonies formed 7 days post infection; scale bar, 100 µm. C, Percentage of infected MEFs at D1 as indicated by GFP fluorescence. MEFs: mouse embryonic fibroblasts, iPSCs: induced pluripotent stem cells, WT: wild type, KO: knock out.

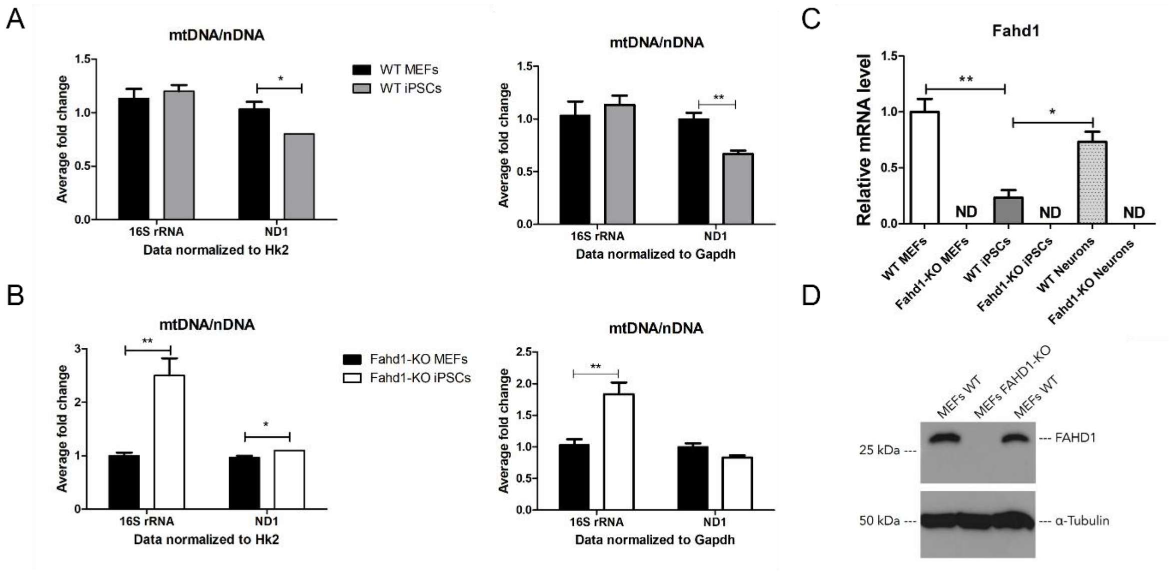

Figure S2: Mitochondrial DNA and Fahd1 expression in MEFs, iPSCs and neurons. (A, B) Average fold change of mtDNA/nDNA ratio showing the expression of the mt genes 16S rRNA and ND1 as normalized to 2 housekeeping genes Gapdh and Hk2. The DNA copy number of these genes was determined in iPSCs and normalized to the expression in MEFs, n=3; (C) Relative mRNA expression of Fahd1 in WT and Fahd1-KO MEFs, iPSCs and Neurons. The expression levels were normalized to WT MEFs, n=3; (D) Western Blot of Fahd1 in WT and Fahd1-KO MEFs showing the absence of the protein in the KO. *p<0.05, **p<0.01. MEFs: mouse embryonic fibroblasts, iPSCs: induced pluripotent stem cells, WT: wild type, KO: knock out, mt: mitochondrial, n: nuclear, ND: not detected.
